# Nintedanib in idiopathic and secondary pleuroparenchymal fibroelastosis

**DOI:** 10.1186/s13023-021-02043-5

**Published:** 2021-10-09

**Authors:** Mouhamad Nasser, Salim Si-Mohamed, Ségolène Turquier, Julie Traclet, Kaïs Ahmad, François Philit, Philippe Bonniaud, Lara Chalabreysse, Françoise Thivolet-Béjui, Vincent Cottin

**Affiliations:** 1 Department of Respiratory Medicine, National Coordinating Reference Center for Rare Pulmonary Diseases, Louis Pradel Hospital, Hospices Civils de Lyon, 28 avenue Doyen Lepine, 69677 Lyon, France; 2 Radiology Department, Hospices Civils de Lyon, Lyon, France; 3 CREATIS, CNRS UMR 5220, INSERM U1206, INSA-Lyon, University Claude Bernard Lyon1, Lyon, France; 4 Department of Respiratory Physiology, Hospices Civils de Lyon, Louis Pradel Hospital, Lyon, France; 5 Department of Respiratory Medicine, Croix Rousse Hospital, Hospices Civil de Lyon, Lyon, France; 6 Department of Pulmonary Medicine and Intensive Care Unit, Constitutive Reference Center for Rare Pulmonary Diseases, François Mitterrand Teaching Hospital, Inserm U1231, University Bourgogne-Franche Comté, Dijon, France; 7 Department of Pathology, Louis Pradel Hospital, Hospices Civils de Lyon, Lyon, France; 8 UMR754, INRAE, Université Claude Bernard Lyon 1, Université de Lyon, Lyon, France

**Keywords:** Pulmonary fibrosis, Pleuroparenchymal fibroelastosis, Pulmonary function tests, CT volumetry, Nintedanib, Progression, Prognosis

## Abstract

**Background:**

Pleuroparenchymal fibroelastosis (PPFE) has a variable disease course with dismal prognosis in the majority of patients with no validated drug therapy. This study is to evaluate the effect of nintedanib in patients with idiopathic and secondary PPFE. Patients admitted to a tertiary care center (2010–2019) were included into this retrospective analysis if they had a multidisciplinary diagnosis of PPFE, had been followed-up for 3 months or more, and had lung function tests and chest CTs available for review. Changes in pulmonary function tests were assessed using non-parametric tests and linear mixed effect model. Lung volumes were measured with lobar segmentation using chest CT.

**Results:**

Out of 21 patients with PPFE, nine had received nintedanib, six had received another treatment and another six patients were monitored without drug therapy. Annual FVC (% of predicted) relative decline was − 13.6 ± 13.4%/year before nintedanib and − 1.6 ± 6.02%/year during nintedanib treatment (p = 0.014), whereas no significant change in FVC% relative decline was found in patients receiving another treatment (− 13.25 ± 34 before vs − 16.61 ± 36.2%/year during treatment; p = 0.343). Using linear mixed effect model, the slope in FVC was − 0.97%/month (95% CI: − 1.42; − 0.52) before treatment and − 0.50%/month (95% CI: − 0.88; 0.13) on nintedanib, with a difference between groups of + 0.47%/month (95% CI: 0.16; 0.78), p = 0.004. The decline in the upper lung volumes measured by CT was − 233 mL/year ± 387 mL/year before nintedanib and − 149 mL/year ± 173 mL/year on nintedanib (p = 0.327). Nintedanib tolerability was unremarkable.

**Conclusion:**

In patients with PPFE, nintedanib treatment might be associated with slower decline in lung function, paving the way for prospective, controlled studies.

**Supplementary Information:**

The online version contains supplementary material available at 10.1186/s13023-021-02043-5.

## Introduction

Pleuroparenchymal fibroelastosis (PPFE) is a rare clinicopathological entity initially described in 1992 by Amitani et al*.* in 13 patients presenting with a condition then called idiopathic pulmonary upper lobe fibrosis [1]. It was coined PPFE in 2004 by Frankel et al. who reported 5 cases with a somewhat comparable condition primarily involving the upper lobes of the lung as well as the pleura [2]. PPFE is characterized by upper lobe pulmonary fibrosis and elastosis with pleural thickening and near-normal distant lung parenchyma [3]. It may be idiopathic or secondary to an underlying condition (e.g., stem cell transplantation, chemotherapy especially using alkylating drugs, radiation therapy or lung transplantation) [4, 5]. Whether idiopathic or secondary, PPFE follows a progressive course with dismal prognosis in the majority of cases [6, 7]. No treatment is validated in PPFE, which remains an orphan lung disease.

Experience with antifibrotic drugs, which slow disease progression in patients with fibrotic interstitial lung disease (ILD) including idiopathic pulmonary fibrosis (IPF) [8], systemic sclerosis associated ILD [9], ILD with a progressive fibrosing phenotype [10], and unclassifiable idiopathic progressive ILD [11], is scarce in PPFE. Pirfenidone has been used in one patient with idiopathic PPFE with associated basal usual interstitial pneumonia (UIP), who showed a stabilized lung function, yet, the patient died 6 months later from respiratory failure [12]. Torrisi et al., reported a possible functional and radiological benefit of low dose prednisone (10 mg/day) combined with pirfenidone in 2 patients with PPFE, however interpretation was limited by the short term follow-up (6 months), and initiation of antifibrotic treatment shortly after lung function had started to stabilize [13]. More recently, Sugino et al., found that antifibrotic agents were less efficacious in patients with UIP associated with idiopathic PPFE than in patients with UIP alone [14]. Herein, we report our experience in patients with PPFE treated with the antifibrotic drug nintedanib. Volumetric imaging was used as adjunct to pulmonary function to assess lung volume changes at the lobar level during disease course.

## Results

### Baseline characteristics

In total, 21 patients were diagnosed with PPFE in multidisciplinary discussion during the study period, including nine who had received nintedanib for 3 months or more. Nintedanib was combined with 10 mg/day of prednisone in six patients. PPFE was idiopathic in 17 and secondary to other conditions in 4 patients, including two who had previously received chemotherapy, and two who had undergone hematopoietic stem cell transplantation. One patient had mild autoimmune features, but none was diagnosed with connective tissue disease. Patients were distributed into the nintedanib group (n = 9), the non-nintedanib treatment group (n = 6; pirfenidone alone [n = 2], prednisone associated or not with mycophenolate mofetil [n = 4]), and the surveillance/no treatment group (n = 6, who were managed with surveillance [n = 5], or received nintedanib for less than 3 months [n = 1]).

Clinical characteristics, usual blood tests, autoantibodies, telomerase gene-related mutations, and high-resolution computed tomography (HRCT) findings were generally comparable between groups (Table 1). In the nintedanib group, definite or probable UIP of the lung bases was present on HRCT and/or lung pathology in 3 out of 9 patients (33%); one patient had a pattern suggestive of organizing pneumonia and another an unclassifiable pattern of interstitial pneumonia in the lung bases. In the non-nintedanib treatment group, no patient had a UIP pattern, but one had a pattern of radiologic nonspecific interstitial pneumonia in the lung bases (he received mycophenololate and low-dose prednisone); in the surveillance group, 2 of 6 patients (33%) had a UIP pattern. There was no significant difference between groups in distinct HRCT measurements except for the transverse tracheal diameter, which was higher in the nintedanib group than in other groups (Table 1). All 6 patients who underwent surgical lung biopsy had definite PPFE on histopathology. Lower lung biopsy found UIP in two, emphysema in two and unclassifiable and non-specific interstitial pneumonia in one each. One patient had chronic pneumothorax secondary to bronchopleural fistula post-surgical lung biopsy. There was no case of spontaneous pneumothorax, mediastinal emphysema or pulmonary mycosis.

**Table 1:** Baseline characteristics by patient group (nintedanib, non-nintedanib, and surveillance)

Parameters (Mean)	Nintedanib (N = 9)	Non-nintedanib (N = 6)	Surveillance (N = 6)	P value
Age (years)	53 ± 24	60 ± 13	55 ± 15	NS
Female	4 (44%)	2 (33%)	5 (83%)	NS
*Smoking history*				NS
Never smoker	5	1	5	
Ex- smoker	4	4	1	
Current smoker	0	1	0	
BMI (kg/m^2^)	17.61 ± 2.21	20.79 ± 3.1	20.64 ± 2.82	NS
Clubbing (No)	9 (100%)	6 (100%)	6 (100%)	NS
Platythorax (yes)	5 (55%)	4 (66%)	3 (50%)	NS
Idiopathic (yes)	7 (77%)	5 (83%)	5 (83%)	NS
ANA +	3 (33%)	4 (66%)	4 (66%)	NS
Tested for mutation (yes)	4 (44%)	3 (50%)	4 (66%)	NS
TERT +	0 (0%)	1 (16%)	2 (33%)	NS
FVC (%)	50 ± 20	57 ± 18	72 ± 23	NS
FEV1/FVC	0.87 ± 0.07	0.87 ± 0.09	0.97 ± 0.08	NS
TLC (%)	63 ± 17	68 ± 14	75 ± 25	NS
RV/TLC (%)	166 ± 57	123 ± 24	112 ± 22	NS
DLCO %	46 ± 19	59 ± 18	48 ± 13	NS
KCO %	83 ± 24	72 ± 9	81 ± 13	NS
Tracheal surface (mm)	397 ± 108*	367 ± 93	211 ± 53	0.009
Lower lobes UIP on HRCT/pathology	3 (33%)	0 (0%)	2 (33%)	NS

ANA: antinuclear antibodies, BMI: body mass index, DLco: diffusion lung capacity for carbon monoxide, FEV1: forced expired volume in 1 s, FVC: forced vital capacity, HRCT: high resolution computed tomography, KCO: transfer coefficient of carbon monoxide, TERT: telomerase reverse transcriptase, TLC: total lung capacity, UIP: usual interstitial pneumonia

### Annualized change in lung function before treatment

During the surveillance period, FVC decreased by − 13.6 ± 13.4% of predicted value (absolute change)/year in the nintedanib group and by − 5.4 ± 13.3%/year in the non-nintedanib treatment group. However, FVC increased by + 0.4 ± 7.0% of predicted in the surveillance group. Similarly, total lung capacity decreased more in the nintedanib group (− 11.1 ± 7.1% of predicted)/year than in the non-nintedanib treatment group (− 2.0 ± 2.4% of predicted)/year and in the surveillance group (− 0.26 ± 4.24% of predicted/year) (Table 2).

**Table 2:** Changes in pulmonary function and body mass index by patient group

LFTs annual change/body mass index	Nintedanib group (N = 9)			Non-nintedanib group (N = 6)			Non-treated group (N = 6)
	Pre-treatment	On treatment	P value	Pre-treatment	On treatment	P value	At diagnosis
Follow-up duration (days)	376 ± 370	530 ± 382	–	1904 ± 2296	593 ± 389	–	1526 ± 1291
Δ FVC (mL)	− 274 ± 188	− 169 ± 214	0.023*	− 193 ± 463	− 275 ± 607	NS	− 50 ± 144
Absolute Δ FVC (%)	− 13.6 ± 13.4	− 1.6 ± 6.02	0.014*	− 5.4 ± 13.3	− 6.6 ± 14.9	NS	+ 0.4 ± 6.97
Relative Δ FVC (%)	− 20.6 ± 16.9	− 6.04 ± 14.5	0.021*	− 13.2 ± 34	− 16.6 ± 36.2	NS	+ 0.7 ± 11.8
Δ TLC (%)	− 11.1 ± 7. 1	− 1.9 ± 9.5	NS	− 2.0 ± 2.4	− 10.7 ± 11.3	NS	− 0.3 ± 4.2
Δ RV/TLC (%)	+ 10.3 ± 18.9	+ 5.5 ± 12.2	NS	− 0.5 ± 2.8	+ 6.1 ± 28.7	NS	+ 1.4 ± 15.5
BMI (kg/m^2^)	17.8 ± 2.0	16.7 ± 1.8	0.031*	20.8 ± 3.1	19.8 ± 3.3	0.328	20.6 ± 2.8 at diagnosis; 20.0 ± 2.5 at last follow− up; P: 0.232

BMI: body mass index, DOF: duration of follow-up, FEV1: forced expired volume in 1 s, FVC: forced vital capacity, TLC: total lung capacity. RV: residual volume.*: significant p value of < 0.05

### Nintedanib treatment and annualized change in lung function

Patients were followed for a mean duration of 15.3 ± 11.4 months (range: 0–33 months) before nintedanib and 17.4 ± 12.0 months (range: 4–41 months) on nintedanib treatment. The slope of both absolute FVC and relative FVC annual decline was significantly lower on nintedanib as compared to the pretreatment period. Relative FVC decline was − 20.6 ± 16.9% of predicted/year before treatment and − 6.04 ± 14.5% of predicted/year on nintedanib (p = 0.021) (Table 2).

**Fig. 1:**
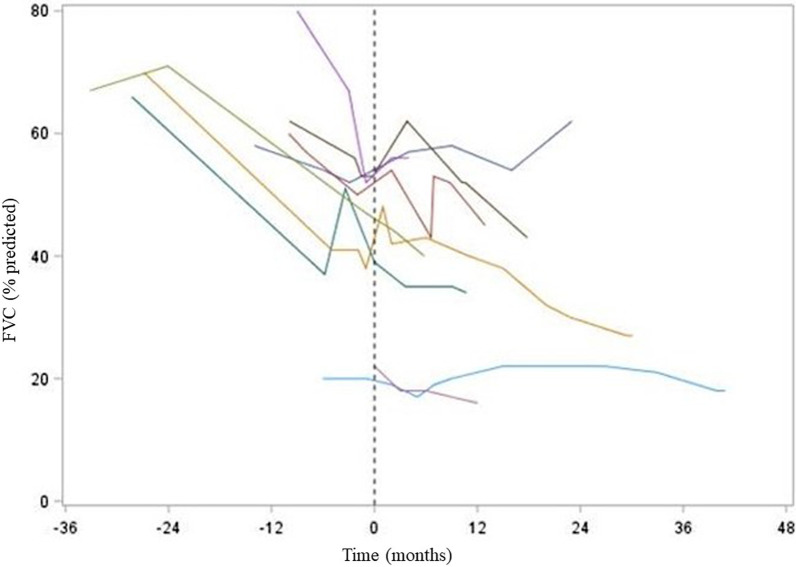
Spaghetti plot representing the evolution of FVC (% of predicted) in 9 patients with PPFE before and on nintedanib treatment. The “0” point and the dotted vertical line represent the initiation of nintedanib. All patients experienced sustained FVC (% of predicted) decline before nintedanib. FVC decline was slowed or stabilized during nintedanib treatment in 8 patients

Using linear mixed model, FVC decreased significantly and consistently by − 0.97% (absolute change) of predicted per month (95% CI: [− 1.42; − 0.52], p = 0.001) during the pretreatment period. At nintedanib initiation, patients had a mean absolute FVC of 41.1% of predicted (95% CI: 29.6–52.3). When nintedanib was initiated, FVC significantly increased by 3.4% of predicted over the first 3 months (95% CI: 0.45; 6.36, p = 0.0250) then started to drop down significantly by − 0.50% of predicted per month (95% CI: − 0.88; − 0.13; p = 0.010) thereafter. The slopes of mean absolute FVC change before and on nintedanib therapy were significantly different (+ 0.47%, 95% CI: 0.16–0.78; p = 0.0037) (Fig. 1).

### Nintedanib treatment and volumetric CT

The changes in particular lobar lung volumes measured by volumetric CT during the surveillance period for the three groups are reported in Additional file 1: Table S1. Among the 21 patients with PPFE, no significant difference was found in the rate of decline in lung volumes measured by volumetric CT, between patients who had or did not have a UIP pattern on imaging (Additional file 1: Table S2).

Among patients who have received nintedanib, the volume of the upper lobes (sum of the right upper lobe volume and of the left upper lobe volume by volumetric CT) decreased by − 233 ± 387 mL/year before treatment initiation, and by − 149 ± 173 mL/year on nintedanib (p = 0.327). The total lung volume (sum of the volume of each of the 5 lobes) decreased by − 415 ± 836 mL/year before treatment initiation, and by − 280 ± 287 mL/year on nintedanib (p = 0.07). Although not statistically significant, there was a trend towards less decline in all lung volume CT measurements during nintedanib treatment (Table 3).

**Table 3:** Changes in lung volumes measured by CT before and on nintedanib treatment (N = 9)

Lung volume measurement	Before nintedanib	On nintedanib	P value
Duration of CT follow-up (days)	361 ± 429 (range: 92–1277)	452 ± 357 (range: 120–1247)	0.575
Δ LUL volume (mL/year)	− 125 ± 211	− 90 ± 125	0.575
Δ LLL volume (mL/year)	− 83 ± 218	− 42 ± 123	0.575
Δ RUL volume (mL/year)	− 107 ± 210	− 58 ± 56	0.484
Δ RML volume (mL/year)	− 36 ± 92	− 12 ± 51	0.889
Δ RLL volume (mL/year)	+ 155 ± 662	− 86 ± 98	0.575
Δ carinal surface/year	+ 9.07 ± 139	+ 52 ± 70	0.401
Δ APDT (mL/year)	− 14.8 ± 21	− 6.5 ± 20	0.401
ΔTDT (mL/year)	− 306 ± 1113	− 2.86 ± 9.23	0.889

APDT: anteroposterior diameter of the thoracic cage, LLL: left lower lobe, LUL: left upper lobe, RLL: right lower lobe, RML: right middle lobe, RUL: right upper lobe, TDT: transthoracic diameter of the thoracic cage

### Safety and tolerability

Tolerability of nintedanib was generally acceptable in the majority of patients, with a median treatment time of 530 ± 382 days. All patients received the full dose at initiation (150 mg twice daily). One patient had diarrhea that was managed successfully with anti-diarrheal drugs. Another patient presented lousy stools properly controlled with diet change. One patient (body mass index [BMI]: 18.9 kg/m^2^) had to stop the drug after seven days because of worsened fatigue on treatment. Another patient (BMI: 15 kg/m^2^) with respiratory failure on continuous non-invasive ventilation discontinued nintedanib after the occurrence of hemoptysis after 976 days of treatment. One patient with a BMI of 20.9 kg/m^2^ who did not tolerate either of the two available dosages of nintedanib and discontinued the drug before 3 months was included into the no treatment group.

All patients had monthly liver function tests during the initial 6-month period and every 3 months thereafter, as recommended, with no abnormality detected. The mean BMI at nintedanib initiation was 17.76- ± 2.03 kg/m^2^. The average BMI of the nintedanib-treated group remained unchanged during the surveillance period, but decreased on nintedanib treatment (p = 0.0315) (Table 2).

Out of 21 patients, ten patients died, with no mortality difference between groups. Three patients received single lung transplantation, two of them died following transplantation (at Day 1 and Month 9, respectively).

## Discussion

Since the inclusion of PPFE in the revised classification of interstitial pneumonias [3], physicians’ awareness of this condition has increased. However, despite improvement in the diagnostic approach, it remains an orphan condition with regards to management, with no treatment approved, and challenging pleural complications. PPFE progresses inexorably in the majority of patients, yet with variable rate [15].

Apart from few reported cases of successful lung transplantation, no treatment has demonstrated efficacy in PPFE [16]. Because intraparenchymal fibrosis is an important histological feature in PPFE, and contrasting results have been reported with pirfenidone in PPFE [6, 12], nintedanib was proposed during multidisciplinary discussion and patients were informed and consented.

All patients with available LFT had lung function deterioration before the initiation of nintedanib with absolute FVC decline of − 0.97% per month (CI 95%: [− 1.42; − 0.52], p = 0.001). On nintedanib, FVC continued to deteriorate but at a slower rate (− 0.50% per month) with an absolute slope change of + 0.47% when compared to the pretreatment period (IC 95%: 0.16–0.78; p = 0.003). Hence, nintedanib was associated with a lower rate of FVC decline (relative reduction, 47%), consistent with the effect of nintedanib in the INPULSIS (1&2), SENSCIS, and INBUILD trials in patients with IPF, SSc-ILD, and PF-ILD, respectively [8–10]. The short-term increment of FVC % following nintedanib initiation might be due to the associated prednisone treatment; whether it is due to a direct effect on lung fibroelastosis or to a transient improvement in the general well-being of patients is unknown.

**Fig. 2:**
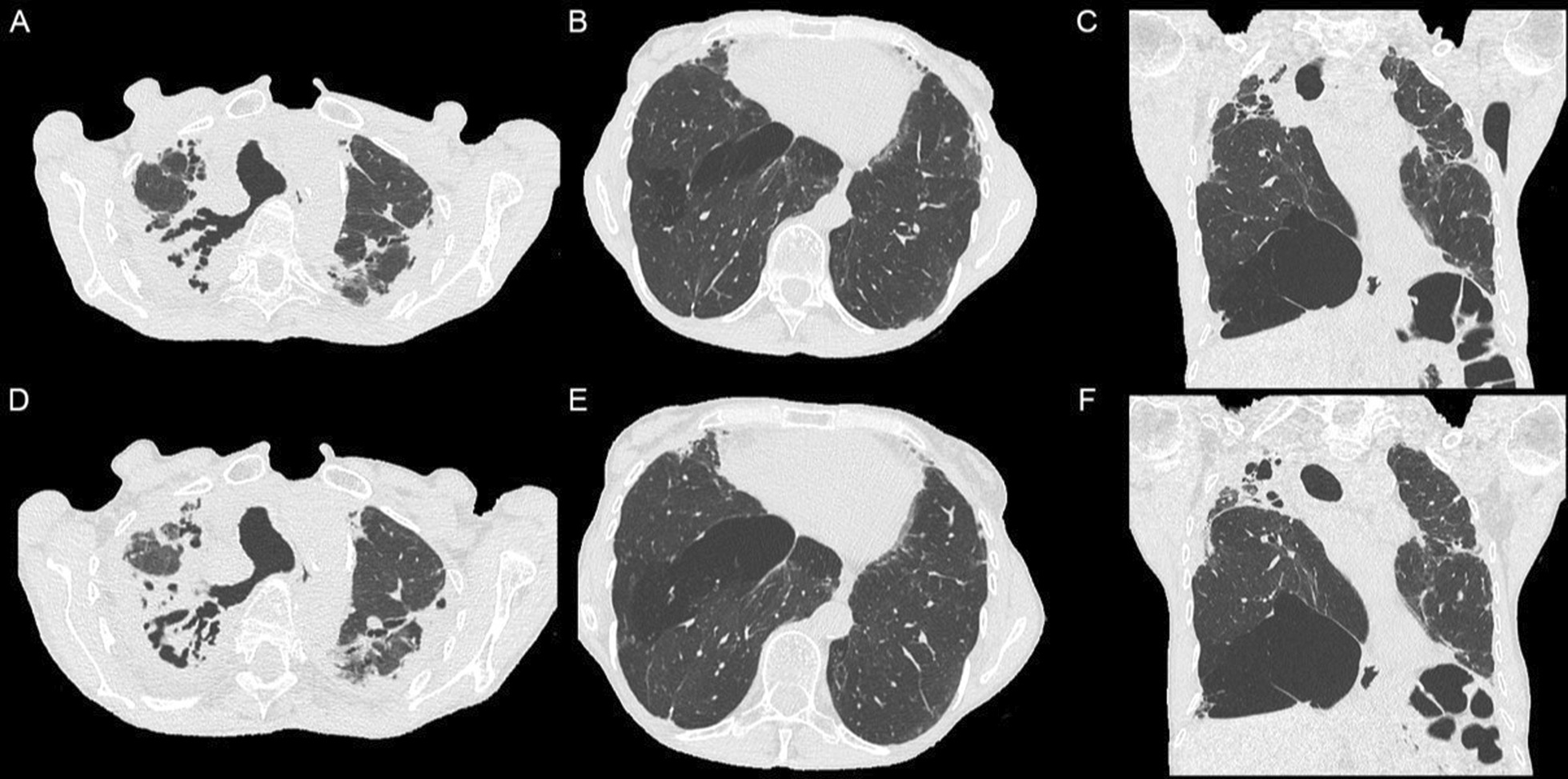
HRCT in a non-smoker patient with PPFE, who received nintedanib as only treatment, demonstrating progression of upper lobe fibrosis (**A** vs **D** and **C** vs **F**), as well as progression of “compensatory” emphysema in the right base (**B** vs **E** and **C** vs **F**)

It is unknown whether FVC is an appropriate marker of disease progression and of treatment effect in PPFE. Changes in FVC may be affected by pleural disease, and by the concomitant presence of UIP in lower lung zones. We therefore measured lobar lung volumes using HRCT in an attempt to assess the net change in upper lobe volume related to PPFE. A trend was observed toward slower volume loss in all lobes, although statistical significance was not reached in this small sample. We observed that the right lower lobe volumes were increasing before nintedanib initiation, and started to decrease on nintedanib therapy. Such paradox might be explained by the development of compensatory lower lobe emphysema following retraction of the upper lobes, a putative mechanism that could have been reduced by nintedanib treatment (Fig. 2). In addition, several patients had more preserved lobules at imaging at the last follow up than at presentation (data not showed), suggesting some air trapping in lower lobes. Air trapping was also reflected by increased residual volume/total lung capacity, especially in the nintedanib group [17].

Although patient demographics at baseline were not statistically different between groups, nintedanib-treated patients tended to have a more severe and more progressive disease prior to treatment. This suggests that treatment was initiated preferentially in subjects with the most active disease. On the other hand, patients who did not receive nintedanib continued to progress on treatment. With disease progression, the upper chest flattened leading to decreased APDT and TDT diameters, whereas the transverse tracheal diameter increased due to outward stretching [18]. At baseline, the transverse tracheal diameter was significantly higher in the nintedanib-treated group, while FVC was lower than in the other groups, consistent with a more severe disease.

Tolerability of nintedanib was comparable to published experience in IPF, despite the relatively low BMI at treatment initiation, and might have been improved by the combination of glucocorticoids and by the young age of our PPFE patients when compared to patients with IPF.

In all cases, the clinical presentations was typical, and the PPFE diagnosis was confirmed in multidisciplinary discussion in all cases, obviating the need of a biopsy in 16 cases [19–21]. Indeed, many groups consider that the risk of pleural complications following surgical lung biopsy may be high in PPFE and no longer perform a biopsy. Patients in our series presented with progressive dyspnea, generalized fatigue, and several had persistent cough. On physical examination, almost half of patients (12/21) had a platythorax, which was confirmed radiologically in all of them. The mean ratio of APDT/TDT was 0.579 ± 0.07 at baseline and decreased alongside with FVC decline in our patients as previously described [18]. Of note, the residual volume/total lung capacity ratio was increased in all patients and was inversely related to APDT/TDT. Whether antifibrotic agents may have an impact on thoracic morphology or mobility is unknown.

## Limitations

The findings presented here are hypothesis generating and should be interpreted with caution, due to the retrospective design, the small sample size, and the relatively short follow-up. Lung function impairment was very severe, and any change would be expected to have a high impact on the patients’ quality of life, which was however not analyzed. Prednisone was used empirically in 6 of 9 patients who received nintedanib, and may have had some effect on the results. However, subgroup analyses based on the ILD morphological pattern and on the use of prednisone or other medications were not possible due to the small sample size. Finally, a survival analysis was not performed due to the limited number of patients in each subgroup.

## Conclusion

In conclusion, this preliminary experience suggests that the potential role of nintedanib to slow down disease progression in patients with PPFE should be further evaluated in prospective, controlled studies.

## Patients and methods

### Patients

We retrospectively reviewed the medical files of all patients admitted to a tertiary referral center between January 2010 and July 2019. Patients were included in the study if they had a multidisciplinary diagnosis of PPFE, and had lung function tests and chest CTs available for review with 3 months follow-up or more. Patients had to have definite PPFE using published criteria [19]. Briefly, definite PPFE was defined pathologically as upper zone pleural fibrosis with subjacent intra-alveolar fibrosis accompanied by alveolar septal elastosis on lung biopsy; and radiologically by pleural thickening with associated subpleural fibrosis concentrated in the upper lobes, with involvement of the lower lobes being less marked or absent on chest CT. Nintedanib (150 mg twice daily) was prescribed off-label for compassionate use at the discretion of the physician, after multidisciplinary discussion, and after obtaining patient’ informed consent.

### Methods

Data on clinical and chest high-resolution computed tomography (HRCT), all available lung function tests (LFT), and outcome, were reviewed. Patients were divided into three groups for analysis: (1) patients who had received nintedanib for 3 months or more (nintedanib group); (2) patients who had received treatments other than nintedanib for 3 months or more (non-nintedanib group); (3) patients who had received no treatment for PPFE or any treatment but for less than 3 months (surveillance/no treatment group). Patients without available clinical, functional or radiological data were excluded. To compare intra and intergroup changes in lung function and volume, surveillance and on-treatment periods were calculated as follows: the surveillance period was the time between the first available LFT/HRCT and the last visit before treatment initiation (for both the nintedanib and non-nintedanib groups), or until the last follow-up visit (for the surveillance group). On treatment period was the time from the first date of nintedanib or non-nintedanib commencement till the last date or last follow-up on treatment. Since patients might not have LFT and HRCT at the same visits, the duration of follow-up was calculated separately for each test. Pulmonary function tests were performed in all patients according to ATS/ERS official statement. Functional residual capacity were measured by Plethysmography and carbon monoxide transfer factor using a single-breath manoeuvre (Medisoft, Belgium) [22].

### CT image analysis

**Fig. 3:**
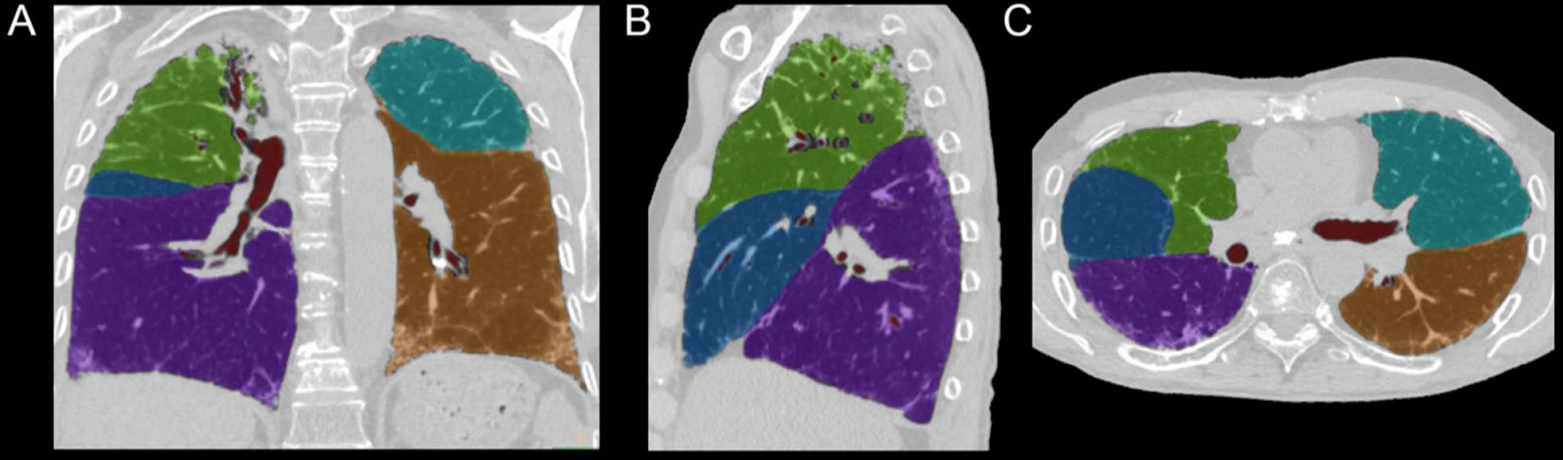
Measurement of lobar lung volume using COPD software (IntelliSpace Portal; Philips Healthcare) of chest HR computed tomography (DECT) images (**A**: coronal, **B**: Sagittal, **C**: axial). A specific color has been attributed for each lobe, and lobar volume was derived

CT images were obtained by full inspiratory HRCT for 2D and 3D volumetric measurements. A radiologist specialized in lung imaging (SSM with 8 years of experience in thoracic imaging) performed a 2D analysis using previously reported parameters, i.e. the transverse tracheal diameter at 1 cm above the carina, the anteroposterior diameter of the thoracic cage (APDT), and the transthoracic transverse diameter of the thoracic cage (TDT) at the level of the fifth dorsal vertebra body scan [18]. A 3D volumetric analysis of the lobar and lung volumes was performed to segment both lung lobes using an appropriate semi-automatic software (COPD, IntelliSpace Portal, Philips) [23]. This software allowed exclusion of the main pulmonary vessels and adjustments to fissure location and lung borders (Fig. 3). Lung volume changes were calculated by subtracting the last measurement from the first measurement. Positive values represented increased lung volume while negative values reflected decrease in lung volume and disease progression in the corresponding lobe. Annual change was then estimated by multiplying the absolute change by 365 and dividing by the time in days between HRCTs. Results were presented in ml/year.

### Statistical analysis

Due to the small sample size of the population (disease rarity) and the expected heterogeneity of disease behavior, normal distribution could not be assumed. Nevertheless, Shapiro–Wilk test was used to check for normal and skewed distributions. Non-parametric tests were used in absence of normality. Continuous variables were presented as mean (proportion) ± standard deviation (SD) using descriptive analysis.

The Wilcoxon signed-rank test was applied to compare pre and post nintedanib changes, and the Mann–Whitney U test was used to compare measures between groups. A linear mixed model was used to evaluate forced vital capacity (FVC) changes before and on nintedanib treatment. No assumption was made during all statistical analysis. Kruskal–Wallis (analysis of variance), Dunn’s multiple comparison analysis and chi-square test were used as appropriate. The level of statistical significance was set at *p* < 0.05 (two tailed). IBM SPSS Statistics for Windows, Version 25.0. Armonk, NY:IBM Corp was used for statistical analysis.

## Supplementary Information

**Additional file 1.** **e-Table 1.** Comparison of changes in lung volumes measured using CT during surveillance period in the three patient groups. **e-Table 2.** Comparison of changes in lung volumes measured by CT between patients with basal UIP vs those without-UIP during the surveillance period.

## Data Availability

The datasets used and/or analysed during the current study are available from the corresponding author on reasonable request.

## Abbreviations

APDT: Anteroposterior diameter of the thoracic cage
BMI: Body mass index
FVC: Forced vital capacity
HRCT: High resolution computed tomography
LFT: Lung function test
PPFE: Pleuroparenchymal fibroelastosis
TDT: Transthoracic transverse diameter of the thoracic cage
TLC: Total lung capacity
UIP: Usual interstitial pneumonia

## References

[1] Amitani R, Niimi A, Kuse F (1992). Idiopathic pulmonary upper lobe fibrosis (IPUF). Kokyu.

[2] Frankel SK, Cool CD, Lynch DA, Brown KK (2004). Idiopathic pleuroparenchymal fibroelastosis: description of a novel clinicopathologic entity. Chest.

[3] Travis WD, Costabel U, Hansell DM (2013). An official American Thoracic Society/European Respiratory Society statement: update of the international multidisciplinary classification of the idiopathic interstitial pneumonias. Am J Respir Crit Care Med.

[4] Beynat-Mouterde C, Beltramo G, Lezmi G (2014). Pleuroparenchymal fibroelastosis as a late complication of chemotherapy agents. Eur Respir J.

[5] Camus P, Von Der Thüsen J, Hansell DM, Colby TV (2014). Pleuroparenchymal fibroelastosis: one more walk on the wild side of drugs?. Eur Respir J.

[6] Watanabe S, Waseda Y, Takato H (2015). Pleuroparenchymal fibroelastosis: distinct pulmonary physiological features in nine patients. Respir Investig.

[7] Watanabe K, Nagata N, Kitasato Y (2012). Rapid decrease in forced vital capacity in patients with idiopathic pulmonary upper lobe fibrosis. Respir Investig.

[8] Richeldi L, Du Bois RM, Raghu G (2014). Efficacy and safety of nintedanib in idiopathic pulmonary fibrosis. N Eng J Med.

[9] Distler O, Highland KB, Gahlemann M (2019). Nintedanib for systemic sclerosis-associated interstitial lung disease. N Eng J Med.

[10] Flaherty KR, Wells AU, Cottin V (2019). Nintedanib in progressive fibrosing interstitial lung diseases. N Eng J Med.

[11] Maher TM, Corte TJ, Fischer A (2020). Pirfenidone in patients with unclassifiable progressive fibrosing interstitial lung disease: a double-blind, randomised, placebo-controlled, phase 2 trial. Lancet Respir Med.

[12] Sato S, Hanibuchi M, Takahashi M (2016). A patient with idiopathic pleuroparenchymal fibroelastosis showing a sustained pulmonary function due to treatment with pirfenidone. Intern Med.

[13] Torrisi SE, Kahn N, Wälscher J (2019). Possible value of antifibrotic drugs in patients with progressive fibrosing non-IPF interstitial lung diseases. BMC Pulm Med.

[14] Sugino K, Ono H, Shimizu H, et al. Treatment with antifibrotic agents in idiopathic pleuroparenchymal fibroelastosis with usual interstitial pneumonia. ERJ Open Res. 2021;7. 10.1183/23120541.00196-2020 PMC7917230 33681342

[15] Yoshida Y, Nagata N, Tsuruta N (2016). Heterogeneous clinical features in patients with pulmonary fibrosis showing histology of pleuroparenchymal fibroelastosis. Respir Investig.

[16] Chen F, Matsubara K, Miyagawa-Hayashino A (2014). Lung transplantation for pleuroparenchymal fibroelastosis after chemotherapy. Ann Thorac Surg.

[17] Kusagaya H, Nakamura Y, Kono M (2012). Idiopathic pleuroparenchymal fibroelastosis: consideration of a clinicopathological entity in a series of Japanese patients. BMC Pulm Med.

[18] Harada T, Yoshida Y, Kitasato Y (2014). The thoracic cage becomes flattened in the progression of pleuroparenchymal fibroelastosis. Eur Respir Rev.

[19] Reddy TL, Tominaga M, Hansell DM (2012). Pleuroparenchymal fibroelastosis: a spectrum of histopathological and imaging phenotypes. Eur Respir J.

[20] Kushima H, Hidaka K, Ishii H (2016). Two cases of pleuroparenchymal fibroelastosis diagnosed with transbronchial lung biopsy. Respir Med Case Rep.

[21] Oda T, Ogura T, Kitamura H (2014). Distinct characteristics of pleuroparenchymal fibroelastosis with usual interstitial pneumonia compared with idiopathic pulmonary fibrosis. Chest.

[22] Pellegrino R, Viegi G, Brusasco V (2005). Interpretative strategies for lung function tests. Eur Respir J.

[23] Si-Mohamed S, Moreau-Triby C, Tylski P (2020). Head-to-head comparison of lung perfusion with dual-energy CT and SPECT-CT. Diagn Interv Imaging.

