# Supplementary material for: Nintedanib in idiopathic and secondary pleuroparenchymal fibroelastosis
Source: Orphanet J Rare Dis. 2021 Oct 9;16:419. doi: 10.1186/s13023-021-02043-5 (PMC8501734; doi:10.1186/s13023-021-02043-5)
Supplement: Supplementary file 1 — Additional file 1. e-Table 1. Comparison of changes in lung volumes measured using CT during surveillance period in the three patient groups. e-Table 2. Comparison of changes in lung volumes measured by CT between patients with basal UIP vs those without-UIP during the surveillance period. [file 13023_2021_2043_MOESM1_ESM.docx]

**Electronic supplemental material**

**e-Table 1.** Comparison of changes in lung volumes measured using CT during surveillance period in the three patient groups.

| **Annual lung volume change** | **Nintedanib group**  **(N=9)** | **Non-nintedanib group (N=6)** | **Surveillance group (N=6)** | **P value** |
| --- | --- | --- | --- | --- |
| Duration of CT follow-up (days) | 361 ± 429 | 1255 ±867 | 735 ±718 | 0.026* |
| Δ LUL volume (mL/yr) | -125 ± 211 | -31 ±138 | -58 ±85 | 0.370 |
| Δ LLL volume (mL/yr) | -83 ± 218 | -34 ±40 | -129 ±213 | 0.456 |
| Δ RUL volume (mL/yr) | -107 ± 210 | -47 ±56 | -45 ±84 | 0.121 |
| Δ RML volume (mL/yr) | -36 ± 92 | -22 ±26 | -58 ±124 | 0.993 |
| Δ RLL volume (mL/yr) | +155 ±662 | -63 ±69 | -76 ±204 | 0.532 |
| Δ carinal surface/yr | +9.07 ± 139 | 27 ±34 | -4 ±43 | 0.519 |
| Δ APDT (mL/yr) | -14.8 ± 21 | -2.28 ±4.54 | -6 ±19 | 0.357 |
| ΔTDT (mL/yr) | -306 ±1113 | -2.93 ±3.41 | -13479 ±32984 | 0.846 |

**Abbreviations:** APDT: anteroposterior diameter of the thoracic cage, LLL: left lower lobe, LUL: left upper lobe, RLL: right lower lobe, RML: right middle lobe, RV: residual volume, RUL: right upper lobe, TDT: transthoracic diameter of the thoracic cage.

**e-Table 2.** Comparison of changes in lung volumes measured by CT between patients with basal UIP vs those without-UIP during the surveillance period.

| Lung volume measurement | No-UIP (N=16) | UIP (N=5) | P value |
| --- | --- | --- | --- |
| Δ LL volume (mL /yr) | -141 ±303 | -292 ±199 | 0.343 |
| Δ RL volume (mL/yr) | -143 ±397 | -290 ±187 | 0.444 |
| Δ LUL volume (mL/yr) | -90 ±191 | -66 ±30 | 0.559 |
| Δ LLL volume (mL/yr) | -38 ±163 | -226 ±200 | 0.070 |
| Δ RUL volume (mL/yr) | -72 ±176 | -83 ±54 | 0.687 |
| Δ RML volume (mL/yr) | -46 ±106 | -22 ±12 | 0.622 |
| Δ RLL volume (mL/yr) | +114 ±520 | -185 ±175 | 0.070 |
| Δ carinal surface/yr | +1.51 | +28 | 0.687 |
| Δ APDT (mL/yr) | -6.9 ±14 | -16 ±29 | 0.823 |
| ΔTDT (mL/yr) | -5772 ±21596 | -565 ±1513 | 0.444 |
| Duration of CT follow-up (days) | 771 ±780 | 633 ±571 | 0.823 |

**Abbreviations:** APDT: anteroposterior diameter of the thoracic cage, LL: left lung, LLL: left lower lobe, LUL: left upper lobe, RL: right lung, RLL: right lower lobe, RML: right middle lobe, RV: residual volume, RUL: right upper lobe, TDT: transthoracic diameter of the thoracic cage, UIP: usual interstitial pneumonia.
